# Autologous Cytokine-Induced Killer Cell Immunotherapy Enhances Chemotherapy Efficacy against Multidrug-Resistant Tuberculosis

**DOI:** 10.1155/2022/2943113

**Published:** 2022-03-17

**Authors:** Peijun Tang, Xingnian Chen, Junchi Xu, Yunlong Hu, Zhijian Ye, Xiafang Wang, Yumei Xiao, Xinghua Shen, Jianping Zhang, Yanjun Feng, Cuilin Shi, Xin Yu, Lixian Yi, Xinchun Chen, Binfeng Lu, Ping Xu, Zhongwen Sun, Meiying Wu

**Affiliations:** ^1^Department of Tuberculosis, The Fifth People's Hospital of Suzhou (The Affiliated Infectious Disease Hospital of Soochow University), Suzhou, China 215000; ^2^Department of Medical Technology, Suzhou Vocational Health College, Suzhou, Jiangsu, China 215009; ^3^Department of Clinical Medical Laboratory, Peking University Shenzhen Hospital, Shenzhen, China 518036; ^4^Department of Pathogen Biology, Shenzhen University School of Medicine, Shenzhen, China 518060; ^5^Department of Immunology, University of Pittsburgh School of Medicine, Pittsburgh PA, USA 15261

## Abstract

**Objective:**

Multidrug-resistant tuberculosis (MDR-TB) causes persistent infection and challenges tuberculosis control worldwide. T cell-mediated immunity plays a critical role in controlling *Mycobacterium tuberculosis* (Mtb) infection, and therefore, enhancing Mtb-specific T cell immune responses represents a promising therapeutic strategy against TB. Cytokine-induced killer (CIK) immunotherapy is based on autologous infusion of in vitro expanded bulk T cells, which include both pathogen-specific and nonspecific T cells from patient peripheral blood mononuclear cells (PBMC) into TB patients. Preclinical mouse studies have shown that the adoptive T cell therapy inhibited Mtb infection. However, the efficacy of CIK immunotherapy in the treatment of MDR-TB infection has not been evaluated in clinical trials.

**Methods:**

We performed a retrospective study of MDR-TB patients who received CIK immunotherapy in combination with anti-TB chemotherapy and those who had standard chemotherapy.

**Results:**

Our results showed that CIK immunotherapy in combination with anti-TB chemotherapy treatment increased the conversion rate of sputum smear and Mtb culture, alleviated symptoms, improved lesion absorption, and increased recovery. The kinetics of serology and immunology index monitoring data showed good safety profiles for the CIK treatment.

**Conclusion:**

Our study has provided strong evidence that CIK immunotherapy in combination with anti-TB chemotherapy is beneficial for MDR-TB patients. A multicenter clinical trial is warranted to evaluate CIK as a new immune therapy for MDR-TB.

## 1. Introduction

Tuberculosis (TB) is the ninth leading cause of death worldwide and the leading cause of death from a single infectious agent, ranking above HIV/AIDS. In 2020, there were an estimated 5.8 million newly diagnosed cases with TB, and this was reported worldwide [1]. The emergence of MDR-TB is a clinical challenge, as its cure rate is extremely low even using combination regimens that include anti-TB first-line and second-line chemotherapy drugs [2].

Since the host immunity plays a critical role in controlling TB, immunotherapies that enhance cellular immune responses against TB should, in principle, be effective for the treatment of MDR-TB. Cytokines, including IFN-*γ*, IL-2, and GM-CSF, have been evaluated for this purpose as they promote the immune response to TB. However, the clinical experiences have demonstrated that these cytokines are not highly effective for MDR-TB treatment [3–5], indicating that alternative and more effective immunotherapeutic strategies are needed.

An alternative strategy to enhance host immunity against TB is adoptive T cell therapy. One type of adoptive T cell therapy is called cytokine-induced killer (CIK) cell therapy, which is being evaluated as an immunotherapy for cancer [6]. CIK cells are generated *in vitro* by stimulating peripheral blood lymphocytes with an anti-CD3 monoclonal antibody (mAb), IL-2, IL-1*α*, and IFN-*γ* [7], resulting in expansion of effector CD4^+^ and CD8^+^ T lymphocytes in bulk. Both antigen-specific and nonspecific T cells are expanded using this protocol. Mouse experiments showed that adoptive transfer of TB antigen-specific T cells inhibited TB infection [8–11]. In human infectious diseases, administration of the CIK cells in an autologous manner decreased the serum HBV load and improved liver function [12]. However, the effect of CIK immunotherapy treatment on MDR-TB remains unknown.

We hypothesized that CIK immunotherapy may also be effective in the context of MDR-TB. Here, we performed a retrospective study comparing the clinical responses of patients treated with CIK immunotherapy in combination with anti-TB chemotherapy to control patients, who were treated with chemotherapy alone. Our goal was to evaluate whether CIK can improve the clinical outcome of MDR-TB patients and provide clinical evidence to support larger clinical trials for CIK immunotherapy.

## 2. Materials and Methods

### 2.1. Patients and Study Design

We analyzed data from patients with MDR-TB who were enrolled in a clinical trial at our hospital (Table 1, clinical trial number: ChiCTR-INR-17012369). The study was approved by the Institutional Review Board of the Affiliated Suzhou Fifth People's Hospital. All patients provided written informed consent. These patients received the routine anti-MDR-TB regimen plus autologous CIK cell transfusion. The patients used as the control were our regular MDR-TB patients who received a routine anti-MDR-TB regimen following the instructions of the Chinese association [13] of anti-TB drug treatment. The criteria for inclusion in this analysis were as follows: (1) sputum smear positive for acid fast staining, (2) sputum culture positive for Mtb and drug sensitivity test-confirmed MDR-TB 6 months before treatment, (3) chest X-ray suggestive of pulmonary tuberculosis, and (4) consent to participate in the study. Patients with MDR-TB were excluded from the trial based on the following: (1) allergies; (2) diabetes and uncontrolled blood sugar levels; (3) severe cardiovascular, liver, kidney, or blood system diseases or any other serious disease that affects chance of survival (such as tumor); (4) clinically significant abnormal ECG (for male patients: prolonged QT interval > 430 ms; for female patients: prolonged QT interval > 450 ms); (5) ongoing drug treatment that might interfere with the trial therapies; (6) mental illness or severe neurosis; (7) poor treatment compliance; (8) pregnancy (or preparing for pregnancy) or lactation; (9) involvement in another clinical trial within the past year; (10) HIV antibody positive, AIDS, or other coinfections; (11) miscellaneous reasons such as a history of drug abuse; or (12) extrapulmonary tuberculosis and nontuberculous mycobacteria infection. In total, 9 patients from the clinical trial and 9 control patients were included in this analysis.

### 2.2. Generation of CIK Cells

Autologous CIK cells were prepared as previously described [14]. Briefly, peripheral blood mononuclear cells (PBMCs) from participants were obtained from whole blood by centrifugation over Ficoll-Hypaque density gradient (Ficoll-Paque Plus; Amersham Biosciences). The PBMCs (2 × 10^6^ cells/mL) were incubated in fresh RMPI-1640 medium (HyClone, USA) containing 10% serum and 1000 U/mL recombinant human gamma-interferon (IFN-*γ*, PeproTech, USA). After incubation for 24 h, anti-CD3 antibody (100 ng/mL, BioLegend, USA), recombinant human interleukin- (IL-) 1*α* (100 U/mL, PeproTech, USA), and recombinant human IL-2 (1000 U/mL, PeproTech, USA) were added to the medium. Cells were incubated and fed every 2 days in fresh complete medium supplemented with rhIL-2 (1000 U/mL) and maintained at a density of 2 × 10^6^ cells/mL.

### 2.3. Analysis of CIK Cell Subpopulations

Cells incubated *in vitro* were collected and stained with anti-CD3-PE-Cy5, CD8-PE, CD3-FITC, and CD56-PE (eBioscience, USA) for 30 min at 4°C. The cells were then analyzed using the BD FACSCalibur flow cytometer and FACS Diva software (BD Biosciences).

### 2.4. Chemotherapy with CIK Transfusion

The anti-MDR-TB chemotherapy regimen followed the WHO “planning and management of drug-resistant tuberculosis” guidelines [15]. The regimen included five groups of drugs: pyrazinamide (Z), amikacin (Am) or capreomycin (Cm), moxifloxacin (Mfx) or gatifloxacin (Gfx), protionamide (Pto) or salicylic acid (PAS), and amoxicillin/clavulanate potassium (Amx/Clv) or clarithromycin (Clr). CIK cells were retrieved when the cell number reached >1 × 10^9^ and CD8^+^ T cell population > 65%. Before the transfusion, the CIK cells were centrifuged at 300 g for 10 min, washed twice with physiological saline, and then incubated with 100 g/L human serum albumin (Shanghai Cuisine) and 1 × 10^6^ U recombinant human IL-2 in 100 mL physiological saline. Each experiment used 1 × 10^9^ cells: CIK cells were administered by intravenous infusion on alternate days for a total of 6 days (3 infusions), 2 times per month, up to 3 months. Before transfusion, the endotoxin content and mycoplasma contamination in the cell product and cell viability were all tested.

### 2.5. Treatment Monitoring

Clinical symptoms and sputum acid stain smears were monitored at 1 day before receiving the 1^st^ treatment and at 1 and 3 months after the day receiving the 1^st^ treatment. Computed tomography (CT) examination was performed every month. The safety index based on routine blood, urine, liver, and kidney function, electrocardiography, and visual acuity was reviewed every week after the day receiving the 1^st^ treatment. Symptoms including coughing, expectoration, and mental state were followed up, and chest CT results were evaluated by two doctors. Evaluation criteria are shown in Table 2.

### 2.6. Statistical Analyses

The data were summarized as the median, mean, and range as applicable and were analyzed using GraphPad Prism version 7.0 (GraphPad Software Inc.). The Wilcoxon matched-pair *t*-test was used to compare data from the same individuals. The nonparametric chi-square test was used to compare the variable response rates between two groups. The Spearman correlation analysis was performed between two parameters. For all tests, a *P* < 0.05 was considered statistically significant.

## 3. Results

### 3.1. Baseline Parameters of the Study Population

This retrospective study included 18 patients with MDR-TB pulmonary tuberculosis: nine were treated with anti-TB chemotherapy alone, and nine were treated with CIK cells plus chemotherapy. The two anti-TB regimens were comparable. We found no significant differences between the two groups in terms of the baseline clinical parameters, including age, gender, body weight, clinical symptoms, sputum examination, and Mtb culture (Table 3). By chest CT, we detected a more severe right pulmonary lesion in the anti-TB chemotherapy-only group compared to the anti-TB chemotherapy plus CIK cell group (*P* < 0.05; Table 3). In addition, we found significant lower UA level and higher frequencies of CD8^+^CD28^+^/CD3^+^ cells in the chemotherapy-only group compared to the anti-TB chemotherapy plus CIK cell group (*P* < 0.05). We found no significant differences in other immunological indexes or serological examinations between the two groups (Table 4).

### 3.2. CIK Treatment Increased the Effect of Anti-TB Chemotherapy

**Figure 1:**
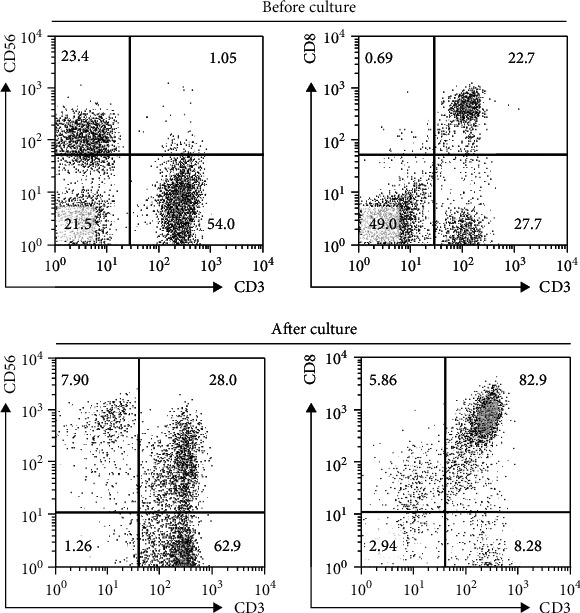
Typical results of frequency of CIK cell subpopulations before and after culture. Before and after cell culture for induction of CIK cells, the frequency of CD3^+^CD56^+^ and CD3^+^CD8^+^ populations was determined by flow cytometry.

**Figure 2:**
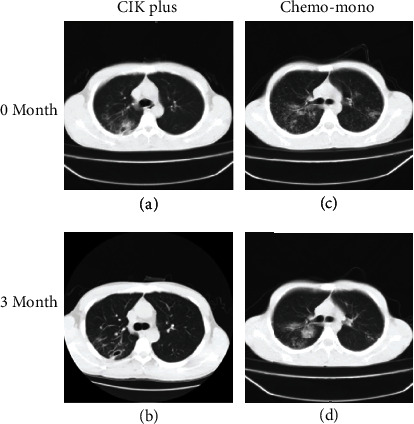
Typical CT results of the lung before and after treatment in Mtb patients: (a) combined CIK cell treatment group before treatment; (b) combined CIK cell treatment group at 3 months after treatment with the same tuberculosis patient in (a); (c) anti-TB chemotherapy-only group before treatment; (d) anti-TB chemotherapy-only group before treatment at 3 months after treatment with the same tuberculosis patient in (c).

Before and after culture, the frequency of CD3^+^CD56^+^ and CD3^+^CD8^+^ populations in CKI cells was analyzed. When the cell number reached >1 × 10^9^ and CD8^+^ T cell population > 65%, CIK cells were retrieved for transfusion (Figure 1). To evaluate the effect of CIK treatment against MDR-TB, we studied the clinical manifestations, the presence of Mtb in sputum by smear staining and culture, and changes in lung lesions by chest CT at 1 day before and 1 and 3 months after the day receiving the first treatment. Compared to the patients treated with anti-TB chemotherapy alone, we observed that symptoms such as cough and expectoration were dramatically alleviated in patients administered with the combined CIK cell treatment at 1 and 3 months after treatment (*P* < 0.05, Table 5). Notably, significantly more patients in the combined CIK cell treatment group achieved conversion of sputum smear (8/9 vs. 2/9) and Mtb culture (9/9 vs. 5/9) than those in the anti-TB chemotherapy-only group (Table 5). Consistently, lung lesions measured by chest CT were significantly diminished in the combined CIK cell treatment group compared to the anti-TB chemotherapy-only group (Figure 2 and Table 5). Interestingly, we noted that body weight significantly increased in patients receiving combined CIK cell treatment but not in those receiving anti-TB chemotherapy only (Table 6). Together, these data indicate that CIK cell treatment is beneficial for MDR-TB patients in terms of facilitating Mtb clearance and recovering lung damage and body weight.

### 3.3. The Kinetics of Serology and Immunology Index Change after CIK Cell Treatment

To evaluate the safety of CIK cell immunotherapy in combination with anti-TB chemotherapy, we monitored the kinetic changes in serology and immunology before and after CIK cell treatment. We found that the proportion of lymphocytes was significantly increased at 1 and 3 months post-CIK cell treatment compared to patients who received anti-TB chemotherapy alone (*P*  < 0.05) (Table 6). Since CD4^+^ T cell subsets exert a critical effect on eradicating *Mycobacterium tuberculosis*, we deduced that CD4^+^ lymphocytes probably contributed to this increase. Notably, the plasma level of IL-2R was significantly decreased in the CIK cell therapy group (*P*  < 0.05) (Table 6), indicating that downregulation of IL-2R might be involved in the anti-TB chemotherapy effect of CIK treatment. There were no significant differences in the frequencies of other blood cells, liver function, renal function, or biochemical analyses between patients receiving the combined treatment and patients receiving anti-TB chemotherapy alone. In addition, we found no differences in the plasma levels of cytokines between the two groups. Interestingly, we noted that the plasma levels of soluble IL-2R were significantly decreased in the patients who received combined CIK cell treatment and significantly lower compared to those receiving anti-TB chemotherapy alone (Table 6).

## 4. Discussion

MDR-TB and extensively drug-resistant tuberculosis (XDR-TB) pose a major challenge for global TB control. Poor treatment outcomes and slow progress in developing and evaluating new TB therapeutics have promoted the development of adjunctive immunotherapy [16]. Here, we took advantage of CIK cell therapy that has shown promise in many disease settings and applied it as an adjunctive immunotherapy together with anti-TB chemotherapy in patients with MDR-TB. We found that patients receiving the CIK cells combined with anti-TB chemotherapy exhibited dramatically alleviated symptoms after one or three months of treatment. In addition, the positive rate of sputum smear and Mtb sputum culture was significantly decreased compared to that in those receiving only anti-TB chemotherapy. The absorption of lesions in those receiving CIK cells was also improved compared to that in the anti-TB chemotherapy-only group. Patients receiving CIK cells showed a significant increase in body weight after 1 month of treatment compared to the anti-TB chemotherapy-only group. This combined treatment protocol elicited no severe adverse effects. Our study strongly supports further clinical trial studies of CIK treatment for TB with larger patient numbers.

Going forward, TB therapeutic development must now consider the immunology of Mtb infection and research should be aimed at delineating the protective immune mechanisms that can be exploited to develop effective adjunct immune therapies [16]. Thus far, biologically and clinically relevant Mtb targets that elicit protective immune responses have not been discovered. Host-directed therapies (HDTs) may be one therapeutic option, particularly for patients with MDR-TB where prognosis is often poor [17]. Targets of HDTs include host factors, such as cytokines, immune checkpoints, immune cell functions, and essential enzyme activities [18]; however, none of these therapies have been shown to be beneficial in controlled clinical trials. Cancer immunology studies have helped shed light on TB immunology, and its underlying principles may guide the development of more effective adjunct treatments.

Patient-derived CIK cells secrete many cytokines, chemokines, and growth factors [19], such as IFN-*γ* and TNF-*α* in patients with MDR-TB with bilateral pulmonary lesions and lung cavitation [20]. It may be hypothesized, therefore, that these secreted factors underlie why and how patients with MDR-TB receiving CIK cell treatment might exhibit improved clinical symptoms. IFN-*γ* is the hallmark of a protective immune response to Mtb infection, although, on its own, it is not sufficient to eliminate the infection. Condos et al. conducted a small-scale trial of the treatment of MDR-TB with aerosolized IFN-*γ* showing that, in the short term, the treatment induced negative sputum conversion and a reduction in cavitary lesions [21]. TNF-*α* production in whole blood may be a specific indicator of sputum conversion at 6 months in patients with MDR-TB [22], and using TNF-*α* inhibitors is accompanied by an increased susceptibility to active TB or reactivation of a latent TB infection [23]. However, the role of the CIK cell secretory profile in patients with TB is, therefore, still unclear and requires further investigation. Interestingly, our data revealed that the plasma level of IL-2R was significantly decreased in the CIK cell therapy group. Previous researches reported that monocytes from TB patients could release soluble IL-2R [24, 25], demonstrating a mechanism of monocyte-mediated suppression of anti-TB T cell responses. Thereby, the effect of CIK on treating TB might be partially dependent on downregulation of IL-2R level.

CIK cells are heterogeneous *ex vivo*-expanded T lymphocytes with mixed T-NK phenotypes and can be expanded from PBMCs cultured with the timed addition of rhIFN-*γ*, anti-CD3 antibody, and rhIL-2. Many case reports, phase 1 clinical trial studies, and retrospective studies supported that CIK cell-based therapy has potential to become a safe cancer immunotherapy [6, 26]. Similar to antitumor immune responses, the anti-TB immunity is also mediated by CD4^+^ and CD8^+^ T cells [27, 28]. In fact, much higher frequencies of TB-specific T cells can be detected in the peripheral blood of TB patients, providing a rich source for cell-mediated therapy. In addition, many preclinical studies have shown efficacy of adoptive T cell therapy for TB [8–11]. Our study clearly supports the feasibility of CIK therapy for MDR-TB. Cytokine-induced killer (CIK) cells have been proven to be a group of heterogeneous cells that are composed of T cells and the non-MHC-restricted natural killer cells [29]. The antigen-presenting cells such as dendritic cells (DCs) can effectively counteract the specificity deficiency of CIK cells and enhance their cytotoxicity [30]. Therefore, the coculture of DCs with CIK cells (DC-CIK cells) may be more valuable to be used as a therapeutic strategy to treat MDR-TB, which remains further exploration.

Our findings suggest that this alternative, combined immune therapeutic strategy for MDR-TB may be clinically effective. It is important to note that the present study is a pilot involving a small number of patients. Further validation studies that enroll a larger cohort are now warranted as well as research into the underlying molecular mechanisms driving the beneficial effects of this treatment protocol.

## Figures and Tables

**Table 1:** Base data of MDR-TB patients.

Type of sample	Age (year)	Gender	Weight (kg)	AFB	Mtb culture	TB DNA	Drug resistance^∗^	Treatment history^#^
CIK plus								
	44	Male	79	1+	Positive	No	SRRSBBBB	AmMfxPtoRfbCfzClrPa
	34	Female	48	3+	Positive	No	RRRRSSRR	AmLfxPtoZPas
	32	Male	65	1+	Positive	No	RRRRRSSS	No treatment
	73	Male	70	4+	Positive	No	RRRRSRSR	PaRfbMfxZPtoClr
	27	Male	55	5+	Positive	No	SRRSSSSS	ZEAmLfxPto
	26	Male	60	1+	Positive	No	RRRRRSRS	PaZPtoClrAm
	59	Male	60	2+	Positive	No	SRSSRSSR	PaRftLfxZ
	62	Female	46	2+	Positive	No	SRRSSSSR	PasEZPto
	25	Female	51	5+	Positive	No	SRRSBBBB	PaRfbMfxZPtoClr
Chemo-mono								
	34	Female	63	1+	Positive	No	SRRSBBBB	HZPasAmLfxPto
	64	Female	52	3+	Positive	No	RRRRBBBB	No treatment
	30	Male	56	4+	Positive	No	RRRSBBBB	ELfxClrPto
	46	Male	50	4+	Positive	No	RRRSSRSR	CmPasLzdCfzAmx/ClvPtoE
	25	Male	62	2+	Positive	No	SRRSBBBB	ZAmLfxPtoPas
	18	Female	36.5	4+	Positive	No	RRRSBBBB	ZAmLfxEPasPto
	66	Male	51	3+	Positive	No	RRRRSSSR	PaRftEZ
	48	Male	65	3+	Positive	No	RRRSRSRR	PasZPtoClrMfx
	24	Male	55	3+	Positive	No	RRRSSSSS	HPasEZPtoAm

^∗^Drug resistance: S—sensitive, R—resistance, and B—blank (not tested). Drug resistance order: SM, INH, RFP, EMB, Pas, Am, Pto, and Lfx. ^#^Antituberculosis drug abbreviation: Am: amikacin; Lfx: levofloxacin; Mfx: moxifloxacin; SM: streptomycin; H/INH: isoniazid; RFP: rifampicin; E/EMB: ethambutol; Pas: para-aminosalicylic acid; Pa: pasiniazid; Pto: protionamide; Rfb: rifabutin; Rft: rifapentine; Cm: capreomycin; Lzd: linezolid; Amx/Clv: amoxicillin and clavulanate potassium; Cfz: chloroformin; Clr: clarithromycin; Z: pyrazinamide.

**Table 2:** The criteria for evaluation of the symptoms or signs in patients.

Symptoms or signs	Criteria for evaluation and score			
	Class 0 (score 0)	Class 1 (score 1)	Class 2 (score 2)	Class 3 (score 3)
Cough	No	Discontinuous coughing during the day, but did not affect work life	Between classes 1 and 3	Frequent coughing or coughing in the day and night and affecting work and sleep
Expectoration	Circadian expectoration < 20 ml	Circadian expectoration < 20 mL-50 mL	Circadian expectoration < 50 mL-100 mL	Circadian expectoration > 100 mL
Hemoptysis	No	Hemoptysis each time < 50 mL	Hemoptysis each time 50-100 mL	Hemoptysis each time > 100 mL
Chest pain	No	Lightly, 2-3 times a day	Severe pain, affecting normal activity, >3 times a day	Persistence, severe pain, and affecting sleep
Dyspnea	No	Little difficulty in breathing in quiet, aggravating in exercise, but did not need oxygen	Uneasy in quiet, intermittent oxygen can be improved	Cyanosis or pale, heart based on class 2, failure or coma, oxygen could not relieve
Weak	No	Light	Medium	Severe
Fever	Normal	<38°C	38-40°C	>40°C

**Table 3:** Baseline characteristics of MDR-TB patients with CIK cell transfusion.

Clinical characteristics	CIK plus	Chemo-mono	*P* value
Sex (*n*, %)			1
Female	3, 33.3	3, 33.3	
Male	6, 66.7	6, 66.7	
Age (years)			0.725
Min–max	25~73	18~66	
Mean ± SD	42.4 ± 18.0	39.4 ± 17.5	
Weight (kg)			0.31
	46~79	36~65	
	59.3 ± 10.8	54.5 ± 8.7	
Symptom (*n*, %)			
Cough	9, 100.0	9, 100.0	1
Expectoration	9, 100.0	9, 100.0	1
Hemoptysis	0, 0.0	3, 33.3	0.206
Chest pain	1, 11.1	1, 11.1	1
Dyspnea	3, 33.3	4, 44.4	1
Fatigue	6, 66.7	3, 33.3	0.347
Fever	2, 22.2	2, 22.2	1
Sputum examination (*n*, %)			
Smear test	9, 100.0	9, 100.0	1
Culture	8, 88.9	9, 100.0	1
CT examination (*n*, %)			
Right upper lung	7, 77.8	8, 88.9	1
Right middle lung	6, 66.7	8, 88.9	0.577
Right lower lung	9, 100.0	4, 44.4	0.029
Left upper lung	8, 88.9	8, 88.9	1
Left lower lung	4, 44.4	7, 77.8	0.335

*n* means score 2 or 3; the other means score 0 or 1. Criteria for evaluation and score are in Table 2.

**Table 4:** Basic data of the serology and immunology test of patients.

Clinical test indexes^∗^	CIK plus (*n* = 9)			Chemo-mono (*n* = 9)			*P* value
	*n*	Mean ± SD	Min~max	*n*	Mean ± SD	Min~max	
WBC (10^9^/L)	9	5.87 ± 1.24	4~7.7	9	5.92 ± 1.61	3.6~8.1	0.936
Hb (10^9^/L)	9	128.78 ± 17.20	100~159	9	118.44 ± 10.22	103~131	0.141
PLT (10^9^/L)	9	182.00 ± 73.55	75~291	9	247.11 ± 88.57	139~399	0.109
N0 (10^9^/L)	9	68.44 ± 10.71	53.4~84.7	9	67.44 ± 12.15	49.9~88.8	0.855
L0 (10^9^/L)	9	20.43 ± 10.57	6.9~41	9	18.19 ± 9.75	5.7~34.8	0.646
ALT5	9	77.67 ± 139.12	3~324	9	13.00 ± 5.89	7~24	0.183
AST5	9	52.67 ± 76.57	9~206	9	17.33 ± 5.72	8~27	0.186
TB (*μ*mol/L)	9	13.20 ± 9.07	4.9~32	9	8.70 ± 2.89	4.7~13.1	0.176
DB (*μ*mol/L)	9	5.24 ± 3.32	1.6~12.2	9	3.39 ± 1.21	1.7~5.1	0.134
ALG (g/L)	9	40.97 ± 5.93	33.9~50.8	9	38.22 ± 3.48	32.5~43	0.248
BUN (mmol/L)	9	4.00 ± 1.17	2.18~6.09	9	3.66 ± 1.17	1.69~5.48	0.554
UA (*μ*mol/L)	9	510.12 ± 148.69	296.6~679.7	9	352.89 ± 135.38	217.8~666.3	0.032
Cr (*μ*mol/L)	9	59.51 ± 11.72	37.8~76.9	9	50.73 ± 12.69	36.9~69.8	0.147
T (%)	9	62.24 ± 17.53	33.3~88	8	70.96 ± 11.12	51.5~85.3	0.247
CD4^+^T (%)	9	35.20 ± 10.30	21~52	8	36.63 ± 8.33	25~48	0.759
CD8^+^T (%)	9	24.31 ± 10.49	11~44	8	31.25 ± 7.34	18~42	0.14
B (%)	9	12.24±4.95	5~23	8	15.31 ± 4.10	10~20	0.187
NK (%)	9	22.16 ± 15.55	953	8	15.44 ± 10.40	335	0.318
CD4/CD8	9	1.63 ± 0.59	0.68~2.38	8	1.22 ± 0.32	0.72~1.78	0.1
L/CD45 (%)	9	24.13 ± 10.83	9.13~38.91	8	22.07 ± 8.69	10.18~36.51	0.673
CD14/CD45 (%)	9	10.42 ± 5.21	4.43~19.95	8	9.29 ± 2.77	6.16~15.14	0.589
CD8^+^CD28^+^/CD3^+^ (%)	8	18.88 ± 6.64	8.51~27.91	8	27.21 ± 6.98	14.75~36.67	0.028
CD4^+^CD28^−^/CD3^+^ (%)	8	3.46 ± 4.11	0.53~13.47	8	1.88 ± 1.90	0~4.58	0.339
CD8^+^CD28^−^/CD3^+^ (%)	8	21.21 ± 14.75	5.92~45.22	8	17.33 ± 8.10	4.27~29.88	0.525
CD4^+^CD28^+^/CD3^+^ (%)	8	55.44 ± 12.91	33.78~69.03	8	52.42 ± 6.96	37.87~58.94	0.569
CD4^+^CD25^hi^/CD3^+^ (%)	8	12.33 ± 5.15	5.91~19.97	8	11.54 ± 4.66	6.39~20.82	0.751
IL-1*β* (pg/mL)	6	5.00 ± 0.00	5~5	5	5.00 ± 0.00	5~5	—
IL-2R (U/mL)	6	1053.67 ± 751.20	631~2572	5	1184.8 ± 442.64	701~1852	0.74
IL-6 (pg/mL)	6	11.93 ± 11.93	4~35	5	9.58 ± 5.15	5~18	0.693
IL-8 (pg/mL)	6	10.83 ± 5.19	5~20	5	13.2 ± 7.98	8~27	0.567
IL-10 (pg/mL)	6	5.48 ± 0.80	5~6.9	5	5.00 ± 0.00	5~5	0.214
TNF-*α* (pg/mL)	6	17.27 ± 15.26	6~47.9	5	15.58 ± 6.36	8~24	0.824
PCT (ng/mL)	6	0.11 ± 0.20	0.02~0.51	5	0.05 ± 0.04	0.03~0.13	0.543

^∗^WBC: white blood cell; Hb: hemoglobin; PLT: blood platelet; NO: nitric oxide; LO: liquid oxygen; ALT: alanine aminotransferase; AST: aspartate transaminase; TB: tuberculosis; DB: direct bilirubin; ALG: antilymphocyte globulin; BUN: blood urea nitrogen; UA: urinalysis; Cr: creatinine; L: lymphocyte; PCT: procalcitonin.

**Table 5:** Comparison for the main treatment indexes between CIK plus and chemo-mono groups after treatment 1 month and 3 months.

Clinical syndromes^∗^	Treatment 1 month			Treatment 3 months		
	CIK plus	Chemo-mono	*P* value	CIK plus	Chemo-mono	*P* value
Cough (*n*, %)	0, 0.0	9, 100.00	<0.001	0, 0.0	8, 88.9	<0.001
Expectoration (*n*, %)	0, 0.0	5, 55.6	0.029	2, 22.2	9, 100.0	0.029
Hemoptysis (*n*, %)	0, 0.0	0, 0.0	1	0, 0.0	1, 11.1	1
Chest pain (*n*, %)	0, 0.0	1, 11.1	1	0, 0.0	0, 0.0	1
Dyspnea (*n*, %)	3, 33.3	5, 55.6	0.637	3, 33.3	5, 55.6	0.637
Fatigue (*n*, %)	1, 11.1	3, 33.3	0.637	1, 11.1	3, 33.3	0.637
Fever (*n*, %)	1, 11.1	0, 0.0	1	1, 11.1	0, 0.0	1
Sputum smear test (*n*, %)	1, 11.1	9, 100.00	<0.001	1, 11.1	7, 77.8	0.015
Sputum culture (*n*, %)	1, 11.1	7, 77.8	0.015	0, 0.0	4, 44.4	0.082
Right upper lobe (*n*, %)	7, 77.8	8, 88.9	1	7, 77.8	8, 88.9	1
Right middle lobe (*n*, %)	6, 66.7	8, 88.9	0.577	6, 66.7	9, 100.0	0.206
Right lower lobe (*n*, %)	9, 100.0	5, 55.6	0.082	9, 0.0	5, 55.6	0.082
Left upper lobe (*n*, %)	8, 88.9	8, 88.9	1	8, 88.9	8, 88.9	1
Left lower lobe (*n*, %)	4, 44.4	7, 77.8	0.335	4, 44.4	7, 77.8	0.335
Improvement in the right upper lobe (yes, %)	3, 33.3	0, 0.0	0.206	3, 33.3	1, 11.1	0.577
Improvement in the right middle lobe (yes, %)	1, 11.1	0, 0.0	1	1, 11.1	0, 0.0	1
Improvement in the right lower lobe (yes, %)	2, 22.2	0, 0.0	0.471	6, 66.7	0, 0.0	0.009
Improvement in the left upper lobe (yes, %)	3, 33.3	0, 0.0	0.206	4, 44.4	0, 0.0	0.082
Improvement in the left lower lobe (yes, %)	1, 11.1	0, 0.0	1	1, 11.1	0, 0.0	1

^∗^ *n* means score 2 or 3; the other means score 0 or 1. Criteria for evaluation and score are in Table 2.

**Table 6:** Clinical test indexes between CIK plus and chemo-mono groups after treatment 1 month and 3 months.

Clinical test indexes^∗^	Treatment 1 month			Treatment 3 months		
	CIK plus	Chemo-mono	*P* value	CIK plus	Chemo-mono	*P* value
Weight (kg)	63.30 ± 10.70	52.70 ± 7.60	0.028	61.10 ± 10.49	53.60 ± 7.90	0.104
WBC (10^9^/L)	5.98 ± 1.76	8.71 ± 3.46	0.063	5.97 ± 1.04	8.48 ± 2.74	0.021
Hb (10^9^/L)	133.63 ± 17.69	123.00 ± 15.12	0.202	141.11 ± 9.03	127.67 ± 20.17	0.087
PLT (10^9^/L)	191.88 ± 56.80	251.33 ± 86.46	0.12	196.22 ± 45.85	266.00 ± 84.31	0.044
N0 (10^9^/L)	63.50 ± 9.46	71.69 ± 9.37	0.094	66.68 ± 9.18	68.37 ± 12.08	0.743
L0 (10^9^/L)	23.80 ± 9.51	16.19 ± 7.21	0.081	23.69 ± 8.79	19.17 ± 10.61	0.339
ALT5	19.88 ± 18.29	14.44 ± 7.81	0.429	16.56 ± 11.24	11.00 ± 5.61	0.203
AST5	20.50 ± 11.19	19.33 ± 9.62	0.82	17.22 ± 3.67	16.00 ± 4.18	0.519
TB (*μ*mol/L)	32.89 ± 62.9	7.69 ± 3.66	0.247	12.58 ± 4.84	9.77 ± 4.61	0.225
DB (*μ*mol/L)	5.41 ± 3.04	3.10 ± 1.84	0.074	4.63 ± 1.89	3.40 ± 2.03	0.201
ALG (g/L)	42.11 ± 6.65	38.93 ± 3.18	0.219	43.61 ± 6.13	41.20 ± 4.36	0.351
BUN (mmol/L)	3.63 ± 0.58	4.01 ± 1.06	0.385	4.40 ± 1.71	3.90 ± 0.59	0.417
UA (*μ*mol/L)	519.60 ± 186.72	467.77 ± 170.72	0.559	436.53 ± 182.8	368.08 ± 268.64	0.536
Cr (*μ*mol/L)	58.01 ± 8.65	54.47 ± 9.14	0.426	64.31 ± 13.84	153.64 ± 176.35	0.149
T (%)	64.16 ± 8.05	60.05 ± 24.11	0.72	64.78 ± 13.63	61.15 ± 9.66	0.633
CD4^+^T (%)	29.98 ± 7.03	20.33 ± 1.88	0.129	31.55 ± 7.76	34.33 ± 6.8	0.565
CD8^+^T (%)	31.68 ± 5.17	39.95 ± 22.56	0.414	31.88 ± 6.51	22.5 ± 7.5	0.077
B (%)	9.20 ± 2.78	7.38 ± 3.71	0.498	16.42 ± 9.86	14.17 ± 6.18	0.665
NK (%)	22.79 ± 5.76	26.57 ± 17.59	0.652	15.82 ± 9.29	20.17 ± 10.83	0.531
CD4/CD8	0.98 ± 0.30	0.59 ± 0.28	0.174	0.99 ± 0.14	1.68 ± 0.65	0.073
L/CD45 (%)	26.13 ± 5.88	12.04 ± 5.99	0.036	22.77 ± 2.86	16.67 ± 6.75	0.131
CD14/CD45 (%)	9.95 ± 3.82	3.86 ± 3.97	0.118	9.03 ± 4.37	6.96 ± 1.85	0.325
CD8^+^CD28^+^/CD3^+^ (%)	16.95 ± 3.48	27.83 ± 9.85	0.095	19.26 ± 4.39	21.9 ± 10.37	0.648
CD4^+^CD28^−^/CD3^+^ (%)	4.06 ± 2.95	4.28 ± 1.98	0.93	3.61 ± 2.95	1.68 ± 2.03	0.251
CD8^+^CD28^−^/CD3^+^ (%)	37.21 ± 17.41	38.40 ± 3.31	0.932	29.09 ± 5.98	20.31 ± 14.4	0.289
CD4^+^CD28^+^/CD3^+^ (%)	41.59 ± 13.33	29.06 ± 9.02	0.308	45.84 ± 3.72	55.34 ± 13.12	0.203
CD4^+^CD25^hi^/CD3^+^ (%)	6.36 ± 1.72	4.80 ± 1.15	0.321	7.80 ± 3.09	8.74 ± 2.36	0.598
IL-1*β* (pg/mL)	5.00 ± 0.00	5.00 ± 0.00	—	5.00 ± 0.00	5.67 ± 1.15	0.286
IL-2R (U/mL)	665.00 ± 170.77	1116 ± 586.9	0.188	519.5 ± 124.72	1347.33 ± 257.12	0.002
IL-6 (pg/mL)	6.75 ± 3.69	8.50 ± 3.54	0.609	10.18 ± 9.25	15.57 ± 18.58	0.63
IL-8 (pg/mL)	9.50 ± 4.80	24.50 ± 17.68	0.151	6.50 ± 1.91	17.33 ± 17.04	0.249
IL-10 (pg/mL)	9.00 ± 8.00	5.00 ± 0.00	0.542	6.00 ± 2.00	5.00 ± 0.00	0.437
TNF-*α* (pg/mL)	12.33 ± 3.93	9.50 ± 2.12	0.412	16.20 ± 8.93	17.53 ± 4.72	0.826
PCT (ng/mL)	0.05 ± 0.03	0.07 ± 0.07	0.622	0.08 ± 0.02	0.06 ± 0.02	136

^∗^WBC: white blood cell; Hb: hemoglobin; PLT: blood platelet; NO: nitric oxide; LO: liquid oxygen; ALT: alanine aminotransferase; AST: aspartate transaminase; TB: tuberculosis; DB: direct bilirubin; ALG: antilymphocyte globulin; BUN: blood urea nitrogen; UA: urinalysis; Cr: creatinine; L: lymphocyte; PCT: procalcitonin.

## Data Availability

The data were summarized as the median, mean, and range as applicable and were analyzed using GraphPad Prism version 7.0 (GraphPad Software Inc.). The Wilcoxon matched-pair *t*-test was used to compare data from the same individuals. The nonparametric chi-square test was used to compare the variable response rates between two groups. The Spearman correlation analysis was performed between two parameters. For all tests, a *P* < 0.05 was considered statistically significant.
